# The Book World of Medicine and Science

**Published:** 1895-04-20

**Authors:** 


### The Book World of Medicine and Science.

Intestinal Anastomosis. By F. Holme Wiggin, M.D. Reprinted from the *New York Medical Journal.*

This is a report of a case, to which is added a criticism of Murphy's button, and an appreciation of Maunsell's operation. Not the least interesting portion of this pamphlet is a letter from Dr. Murphy defending his apparatus. Incidentally a point crops up which is of more than local interest. Dr. Murphy points out that he has done his best to ensure that the buttons are properly made, but that there are still defective buttons on the market. This, he says, he is powerless to avoid, as the ethics of the profession prevent him from procuring a patent for the button, and thus controlling its manufacture. Undoubtedly this is a difficulty. Useful inventions are apt to be lost to the world from the disrepute into which they fall from badness of manufacture.

Our State Hospitals. By Thomas M. Dolan, M.D., Leicester. (John Richardson and Co. pp. 66.)

This book is the outcome of an earnest desire to show not merely what ought to be done in the way of improvement of our workhouse infirmaries, but to demonstrate by the results of personal experience what can be done in that direction. Dr. Dolan takes up the position that for the proper treatment of the sick in hospital certain things are fundamental, and that these things are the same whether the hospital is intended for paupers or for non-paupers. He seems, in fact, to somewhat minimise the work done by the general hopitals, and to be inclined to so improve the status of the poor law infirmary, not only actually but also in the eyes of the public, that it should practically meet the hospital requirements of the people. He says, "It is inadvisable to have two hospital systems, one applicable for the very poor, another for a superior class of poor, or one for the middle class sickness being the leveller of all class distinctions." This is not the place to argue this matter, although one may remark that so long as class habits vary so greatly as they do in health, the miseries of sickness will be much aggravated by choosing that as the time for experiments in social equality. Be this as it may, however, one may fairly endorse Dr. Dolan's fundamentals in regard to the hospital treatment of the sick, of whatever class they be. These are: (*a*) Properly-constructed wards, lighted and ventilated on the best principles; (*b*) not less than 1,000 cubic feet of space for each patient; (*c*) baths, lavatories, and water-closets on the most improved principles—isolation of each section, with free access of air within and without; (*d*) certificated nurses of three years' training; and (*e*) medical officers who will give all their time to their true duty, viz., their patients; clerk work, dispensing, and so on to be done by clerks and dispensers. The author of this book has himself been a medical officer to a poor-law infirmary, and is familiar with the administration and planning of such institutions. There are chapters on construction, ward furniture, nursing arrangements, the position and duties of the medical officer, lunacy administration, report writing, dietaries, &c., all of which will be found useful by medical officers, and especially by such guardians and poor-law officials as are anxious to deal well with the sick poor who come under their care. Several illustrations and a number of plans are included in the work before us.

